# Knowledge and Preventive Practices of Hepatitis B Transmission among Dental Students and Interns in a Tertiary Hospital: A Descriptive Cross-sectional Study

**DOI:** 10.31729/jnma.4786

**Published:** 2020-02-29

**Authors:** Durga Bhandari

**Affiliations:** 1 Department of Internal Medicine, Kantipur Dental College Teaching Hospital and Research Center, Kathmandu, Nepal

**Keywords:** *hepatitis B*, *dental students*, *vaccination*

## Abstract

**Introduction:**

Hepatitis B is one of the most common contagious diseases in Nepal and is a significant public health issue. It is transmitted through contact with contaminated blood or other bodily fluids on broken skin or mucous membranes. Junior doctors and dentists are at particular risk of hepatitis B exposure. This study aims to find the level of knowledge of transmission and prevention of hepatitis B among the dental students.

**Methods:**

This was a descriptive cross-sectional study conducted among dental students and interns at Kantipur Dental College Teaching Hospital and Research Center, Kathamndu from January 2019 to February 2019 after ethical approval was provided by the Institutional Review Committee. The study included dental students and graduate intern doctors. Convenience sampling was done. Point estimate at 95% Confidence Interval was done along with frequency and proportion of binary data.

**Results:**

Out of one hundred forty two students, 68 (48%) of participants had completed a full course of hepatitis B vaccine. Thirty seven (26%) had started but had less than three recommended shots and 37 (26%) had not received any vaccines for hepatitis B prevention. Only 14 (10%) of the study group had checked their hepatitis B titer prior to commencing medical education.

**Conclusions:**

There is also a lack of understanding of transmission, prevention and post exposure prophylaxis for hepatitis B infection among them among new health care providers in Nepal. This puts both the practitioners and patients at risk of chronic hepatitis B infection, which is unnecessary given cheap and easy prevention strategies, can virtually eliminate the risk.

## INTRODUCTION

Hepatitis B virus (HBV) is one of the most important transmittable diseases in healthcare settings of Nepal. It is also a major global health problem. HBV is transmitted through percutaneous or mucosal exposure to infectious blood or body fluids. Health care practitioners (HCP) may be exposed to HBV through contaminated medical or dental instruments, needle-stick injuries, or saliva and/or blood contamination on mucosal surfaces or lacerated skin. HBV can be transmitted in the absence of visible blood and remains infectious on environmental surfaces for at least seven days.^1^

HCP’s are at risk of contracting the illness and transmitting it to other patients. An effective vaccination for Hepatitis B has been available since 1982. The vaccine is 95% effective in preventing infection and the development of chronic disease and liver cancer due to hepatitis B.^2^ After an exposure to a known hepatitis B positive source, hepatitis B immunoglobulin in addition to vaccination may be appropriate in the absence of documented vaccination.

This study aims to find the level of knowledge of transmission and prevention of hepatitis B among the dental students.

## METHODS

This study was a descriptive cross-sectional study conducted in Kantipur Dental College Teaching Hospital and Research Center in January 2019 to February 2019. Ethical approval was provided by the Institutional Review Committee of Kantipur Dental College Teaching Hospital and Research Center. The participants of the study included dental students and graduate intern doctors who were provided with a written questionnaire after agreeing to be part of the study. Convenience sampling was done.

Sample size was calculated as,

n = z^2^ × (p × q) / e^2^

   = 1.96 × (0.5 × 0.5) /0.09^2^

   = 119

After taking 10% of non-response rate, the total sample size is taken as 142.

Selection bias and reporting bias has been minimized as much as possible.

Data was collected and managed in Microsoft excel and descriptive analysis were performed. Point estimate at 95% Confidence interval was done along with frequency and proportion for binary data.

## RESULTS

One hundred and forty-two patients were included in the study, 109 (77%) were female and 33 (23%) were male. The average age of the study group was 22 years, with a range of 17-23 years. Among our study group, 49 (35%) were first year students, 15 (11%) were second year students, 17 (12%) were third year students, 26 (18%) were fourth year students, 13 (9%) were final year students, 22 (15%) were intern doctors. Only 68 (48%) respondents had received the full course of hepatitis B vaccinations, and only 14 (10%) of the study group had checked their hepatitis B titer prior to commencing medical education. Thirty-seven (26%) respondents had started but not completed all three doses and 37 (26%) had not received any vaccines for hepatitis B prevention. Therefore 74 (52%) respondents were not protected against HBV.

Overall there was a considerable lack of understanding of the behavior of HBV, despite 142 (100%) of the study sample seeming to understand the mode of transmission. Only 55 (39%) of respondents realized that HBV remains viable outside of the body for seven days. Seven (5%) thought that hepatitis B was not vaccine preventable, and 12% thought that standard safety precautions would not protect against it. When it came to prevention and management of an exposure, only 15 (11%) of respondents selected instrument sterilization as important for prevention and 18 (13%) selected appropriate instrument disposal as important. The highest number of respondents selected ‘consult a doctor’ after a likely exposure, followed by vaccination, and many of them answered that there is no post-exposure treatment. Only 12 (8%) of the participants considered post exposure prophylaxis following exposure, and only 12 (8%) of respondents thought water irrigation was appropriate treatment for an exposure to body fluids. Twelve percent of respondents selected ‘do not know’ to the question: If you sustain a needle or other accidental hepatitis B exposure is there any emergency treatment available, and 13% selected the option ‘there is no treatment available’.

**Table 1:** Knowledge about Hepatitis B.

Variable	True n (%)	False n (%)
A person with active Hepatitis B is safe to be performing invasive medical or dental procedures.	19 (13%)	123 (87%)
Hepatitis B is infectious and can survive outside of the body for seven days.	55 (39%)	92 (65%)
Hepatitis B can be spread via sexual intercourse, exposure to contaminated blood via needle or sharp exposure or body fluids splashing on broken skin.	142 (100%)	0
Hepatitis B is not vaccine preventable.	7 (5%)	135 (95%)
Universal Safety precautions will not protect you against hepatitis B.	17 (12%)	125 (88%)
Condoms during sexual activity will not protect you against Hepatitis B.	19 (13%)	123 (87%)

**Table 2:** If you sustain a needle or other accidental hepatitis b exposure is there any emergency treatment available.

Variable	n (%)
Passive Immunization	12 (8%)
Consult a Doctor	49 (35%)
Vaccine	34 (24%)
Post Exposure Prophylaxis	12 (8%)
I don’t Know	17 (12%)
There is no Treatment	18 (13%)

**Table 3:** If you do sustain a needle stick injury or accidental hepatitis b exposure what should you do next?

Variable	n (%)
Wash hand with alcoholic agent	15 (11%)
Wash the area with running water	12 (8%)
Vaccine	30 (21%)
Visit a Doctor	52 (37%)
Check Antibody Titer	12 (8%)
Blood Test	8 (6%)
PEP	11 (8%)
Sterilize Needle	2 (1%)

**Table 4:** Before performing as invasive medical or dental procedure how can a health worker protect themselves? List three things.

Variable	n (%)
Use of Gloves and Face Mask	121 (85%)
Vaccination	126 (89%)
Wash Hands Properly	98 (69%)
Use of Sanitizers	25 (18%)
Careful Procedures	21 (15%)
Safe Disposal of Infected Instruments	19 (13%)
Sterilized Instruments	16 (11%)

## DISCUSSION

The findings of this study demonstrate three things. Firstly: that vaccination levels among future dentists and doctors in Kathmandu at this college are inadequate, secondly, that students do not have a good understanding of how to prevent HBV transmission, and thirdly that they do not understand how to appropriately manage a potential infectious body fuid or blood exposure if it occurs.

Needle stick or other sharps injuries are one of the most common injuries for dentists and doctors. The incidence of transmission of HBV via needle stick injury in one study was approximately 2% with Hepatitis B, depending on the Hepatitis B e Antigen (HBeAg) status of the source individual.^3,4^

Hepatitis B vaccination is recommended for any exposure regardless of the source person’s HBV status. HBIG may be recommended depending on the source person’s infection status, host vaccination status and, if vaccinated, serological response to the vaccine.^5,6^

Vaccination remains the mainstay of HBV prevention efforts. Only 48% of the participants in our study had completed the full Hepatitis B vaccination schedule. In a similar study among study among dental students in Karnataka, India,^7^ 64% were immunized against hepatitis B, 79.65% in a study of medical and dental students in a Nigerian University^8^ and a similar study performed in medical college in Nepal^9^ had 73.5% students immunized against Hepatitis B vaccine. It was only 41% in another study among dental students done in Saudi Arabia.^10^

As per the current guidelines of CDC, HBV infection does not preclude the practice or study of medicine, surgery, dentistry, or allied health professions.^9^ Standard Precautions (formerly known as universal precautions) should be sufficient to protect both patient and HCP. These include hand washing before and after every patient, using disposable personal protective equipment (gown, gloves, eye and face masks) for every patient, sterilization of instruments and disinfecting clinical surfaces before and after every patient. In the event of significant quantities of body fluids, or known infective status, wearing two pairs of gloves may be additionally protective. These procedures can virtually eliminate HBV transmission between patient and doctor, and patient to patient.

CDC also recommends that in the event of exposure, the exposed individual should wash needle sticks and cuts with soap and water, fush splashes to the nose, mouth, or skin with water and irrigate eyes with clean water, saline, or sterile irrigants. Each institution should have a policy in place for managing body fuid exposures, including organizing for testing of the infectious status of the individual exposed, and the individual whose body fuid did the contaminating, organizing medical consultation regarding post exposure prophylaxis if appropriate, and following up after three months with repeat tests of both individuals to cover the lag time for serology to become positive post infection. Vaccination (3-dose series) should be followed by assessment of hepatitis B surface antibody to determine vaccination immunogenicity and, if necessary, revaccination. Health-care providers who do not have protective concentrations of anti-HBs (>10 mIU/ml) after revaccination (i.e., after receiving a total of 6 doses) should be tested for HBsAg and anti-HBc to determine their infection status.^5^ Only ten percent of the students in this study had checked their anti-HBs titer. Students along with other health care professionals should check their protective titers before starting dental college according to current CDC recommendations.^11^

The questionnaire was administered in English to a group of students for whom English is not their first language. They may not have fully understood the phrasing of the questions. The true and false format may provide false results because by guessing the students had a ffty percent chance of being correct. The sample size was small and confned to one college thus may not be representative of other teaching institutions.

## CONCLUSIONS

This study is an important preliminary step to understanding what graduating HCP’s are being taught about HBV prevention in Nepal. It demonstrated that prevention and post-exposure treatment knowledge was really lacking. Most of the students were unaware of their protective status which makes them more prone to HBV infection. This is a significant public health issue that has an effective and cheap remedy. Safe injection practices, eliminating unnecessary and unsafe injections, can be effective strategies to protect against HBV transmission. Health education and policies regarding preventive measures of Hepatitis B and vaccination should be mandatory learning for all HCP’s. HCP’s need further knowledge about the standard precautions that should be taken before performing any procedure. They also need to know that the vaccination is effective in preventing Hepatitis B and there is post exposure treatment in case of any accidental exposure.

## Conflicts of Interest:

None.
